# The same oculomotor vermal Purkinje cells encode the different kinematics of saccades and of smooth pursuit eye movements

**DOI:** 10.1038/srep40613

**Published:** 2017-01-16

**Authors:** Zongpeng Sun, Aleksandra Smilgin, Marc Junker, Peter W. Dicke, Peter Thier

**Affiliations:** 1 Department of Cognitive Neurology, Hertie Institute for Clinical Brain Research, University of Tübingen, 72076 Tübingen, Germany; 2 Graduate School of Neural and Behavioural Sciences, International Max Planck Research School for Cognitive and Systems Neuroscience, University of Tübingen, 72074 Tübingen, Germany

## Abstract

Saccades and smooth pursuit eye movements (SPEM) are two types of goal-directed eye movements whose kinematics differ profoundly, a fact that may have contributed to the notion that the underlying cerebellar substrates are separated. However, it is suggested that some Purkinje cells (PCs) in the oculomotor vermis (OMV) of monkey cerebellum may be involved in both saccades and SPEM, a puzzling finding in view of the different kinematic demands of the two types of eye movements. Such ‘dual’ OMV PCs might be oddities with little if any functional relevance. On the other hand, they might be representatives of a generic mechanism serving as common ground for saccades and SPEM. In our present study, we found that both saccade- and SPEM-related responses of individual PCs could be predicted well by linear combinations of eye acceleration, velocity and position. The relative weights of the contributions that these three kinematic parameters made depended on the type of eye movement. Whereas in the case of saccades eye position was the most important independent variable, it was velocity in the case of SPEM. This dissociation is in accordance with standard models of saccades and SPEM control which emphasize eye position and velocity respectively as the relevant controlled state variables.

Saccades and smooth pursuit eye movements (SPEM) are two synergistic types of eye movements which allow the visual system to exploit the advantages of foveal vision for the analysis of objects of interest (‘targets’). Whereas saccades shift the target image onto the fovea, SPEM are deployed to stabilize it there despite movements of the target relative to the observer. The kinematics of these two types of goal-directed eye movements differ profoundly. Saccades are fast, short-duration eye movements, reaching peak velocities of many 100°/s. These features ensure that the time during which vision is compromised because of retinal image slip is kept to a minimum. On the other hand, SPEM are continuous eye movements confined to velocities below 10°/s or at most 30–50°/s^1^,^2^,^3^,^4^. To accommodate the very different kinematics of saccades and SPEM, different control strategies are needed. This is why the discussion of their implementation has been dominated by the assumption of specialization and segregation^5^ (for another view see refs 6 and 7). Early work on the oculomotor role of the cerebellum seemed to be in line with the concept of segregation, namely the flocculus/paraflocculus as cerebellar substrate of SPEM^8^ and the oculomotor vermis (OMV) subserving saccades^9^. Yet, it later became clear that lesions of the OMV not only impair saccades^10^,^11^,^12^ but also the initiation of SPEM^13^,^14^,^15^, indicating a role of OMV in both types of eye movements. Correspondingly, electrical stimulation of the OMV not only evoke saccades^16^,^17^ but also pursuit-like slow eye movements^4^. Finally, single-unit recording studies have shown that some Purkinje cells (PC) in the OMV respond to both saccades and SPEM^18^,^19^. Are these ‘dual’ PCs representative of OMV PCs at large or are they oddities with little if any functional relevance? However, if these PC units are representative of the whole population rather than oddities, this would probably suggest that such PCs offer a hitherto unknown functional contribution to an aspect of eye movements shared by saccades and SPEM.

Studies on the role of the OMV in SPEM have suggested that it may play a major role in controlling eye velocity in specific directions. The earliest evidence came from a study of smooth-pursuit of targets moving sinusoidally along the horizontal^20^. The authors of this study reasoned that responses reflected primarily eye velocity because OMV PC simple spike (SS) units increased their modulation with increasing target speed. Later work, employing pursuit targets that moved according to a sinusoidal profile, showed that at least some neurons in the OMV seemed to prefer gaze velocities in directions other than the horizontal^21^. In our previous work on smooth-pursuit- related OMV PC SS units, we explored the kinematic preferences in more detail by modeling the relationship between discharge and the early, open-loop SPEM based on a linear combination of eye acceleration, velocity and position. This analysis clearly indicated that indeed eye velocity is the most relevant kinematic parameter^22^.

Given that velocities of saccadic eye movements are much higher than those of SPEM, a simple linear encoding of eye velocity for both types of eye movements would not work and a coding scheme reflecting the different kinematics of saccades and SPEM should be expected. Moreover, how representative are the ‘dual’ PC SS units? To answer these questions we set out to compare the discharge characteristics of eye movement-related PC SS units recorded from the OMV during saccades and SPEM in the same direction or to saccades and SPEM in 8 directions. We report that many PC SS units are sensitive to saccades and SPEM and, moreover, that different kinematic parameters are emphasized during SPEM and saccades.

## Materials and Methods

### Animals and surgical procedures

Two male rhesus monkeys E and I (Macaca mulatta, males, 10 and 6 years old; purchased from the German Primate Centre, Göttingen, Germany) were subjects in this study. In both monkeys, the recording chamber (diameter 30 mm) was implanted in the sagittal midline, tilted posterior by 20° (monkey E) and 40° (monkey I), respectively, allowing us to explore larger parts of their brainstem. For monkey E, in order to optimally reach the OMV, the chamber was fitted with an adapter reducing the tilt angle by 20°, while in monkey I, an adapter reducing it by 35° was chosen (see Supplementary Fig. S1). The experiments on these animals including the surgical and behavioural protocols were approved by the Regierungspräsidium Baden-Württemberg (Ref. 35; permit-number N6/13), conducted in accordance with the guidelines of the National Institutes of Health for Care and Use of Laboratory Animals and supervised by the veterinary administration (Landratsamt Tübingen (Abt. 32)).

### Behavioural tasks

The monkeys were seated head-fixed 40 cm in front of a CRT monitor in darkness. The eye position measured by eye coil was calibrated using a 9-point calibration that considered linear, quadratic and mixed term dependencies (typical grid size: 30×30°, target diameter: 0.4°). Next they were trained to keep stable eye fixation on the visual target (dot diameter 0.2–0.4°, depending on animal), displayed on a CRT monitor in front of them at a distance of 40 cm. Proper fixation was assumed if the eyes stayed within an invisible rectangular window of 1.5–4° around every displayed target and a trial was aborted if eye position exceeded the limits of this window.

#### Visually guided saccades

The monkeys were required to make precise saccades starting from the straight ahead fixation dot to a peripheral target having the same size and appearance. The peripheral target was presented in 8 possible positions in the frontoparallel plane (rightward: 0°, right-up: 45°, upward: 90°, left-up: 135°, leftward: 180°, left-down: 225°, downward: 270°, right-down: 315°) at constant eccentricities of 10°. The peripheral target appeared after a variable (500–1000 ms) time of fixation at which time the central target was turned off. The transition from the central to the peripheral target was the go signal for the monkey to make a saccade to the peripheral target and to maintain fixation there for at least 300 ms before being allowed to return to straight ahead. A trial was aborted if the monkey did not initiate a saccade within 400 ms after the go signal. The eyes had to stay within an invisible eye position window (1.5–4°) centered on the target independent of its location. This window was transiently turned off during the transition of the target to the periphery. Target directions were randomly chosen from trial to trial.

In many experiments, we kept the saccade target direction constant in order to be able to vary target eccentricity randomly between 10°, 7° and 4°. Here, in the interest of time, the experimenter tested a few trials of up, down, left and right saccades in order to choose a “best” direction from this set by listening to the audiomonitor and inspecting the online records of the spike trains. In these experiments the direction of smooth pursuit eye movements tested (see the following) was the same and likewise constant.

#### Smooth pursuit eye movements (SPEM)

To evoke SPEM with no catch-up saccades in at least the first 200–250 ms after eye movement onset we deployed a step-ramp paradigm^23^. Each SPEM trial started with a variable period of fixation on the target displayed in the central position for 500–1500 ms. Then the target stepped by 1.4–2.4° in a particular direction, a step that was immediately followed by a ramp-like movement of the target at a velocity of 12°/s in the opposite direction. The pursuit directions were chosen from the same set of directions also used for saccades. The step amplitude was adjusted individually for each monkey such that the target ramp would have moved the image of the target back into the fovea at the time of the onset of the smooth-pursuit eye movement, thereby eliminating the need for catch up saccades. The trial duration was always 2400 ms. The eye position control window had a size of 2–4° throughout a trial.

In experiments, in which saccade- and SPEM-related responses were collected in 8 directions, the two types of eye movements were studied in separate blocks whose order changed from neuron to neuron. Within individual blocks, the 8 eye movement directions were presented randomly interleaved. In experiments, in which 10°, 7° and 4° saccades and SPEM were studied in an identical direction, trials were presented randomly interleaved.

### Electrophysiological procedures

Action potentials of PCs were recorded extracellularly using glass-coated tungsten microelectrodes (1–2 MΩ impedance at 1 kHz; Alpha Omega Engineering, Nazareth, Israel) advanced with an 8-probe electrode system (Alpha Omega Engineering, Nazareth, Israel). In most cases we used maximally 4 electrodes, arranged linearly either along the rostrocaudal or the medio-lateral axis and separated by 2 mm each. We approached the OMV by using the stereotaxic coordinates provided by the anatomical MRI scans and identified the OMV by resorting to well established criteria, namely the dense saccade-related granule cell background and the appearance of saccade-related single units in the neighbouring layers. The electrode signal was band-pass filtered for frequencies from 300 to 3000 Hz to enable the isolation of spikes. SS and CS were detected online by using a Multi Spike Detector (Alpha Omega Engineering, Nazareth, Israel) which detects and sorts spikes according to the features of template waveforms.

### Data analysis

All analyses were performed with in-house Matlab programs (The MathWorks Inc., Natick, MA). Saccades were automatically detected by applying an eye velocity threshold of 20°/s. The detection of the onset of SPEM required several computational steps. A first approximation of movement onset was obtained by identifying the time point at which the eye velocity exceeded the mean eye velocity in the first 80 ms after the onset of the target ramp by three standard deviations for 40 consecutive milliseconds. Then two linear regressions were fitted to the eye velocity records - the first one on the eye velocity in the 200 ms before this time point and the second one on the 150 ms to follow. The interception point of the two regression lines was used as final estimate of SPEM onset. SPEM trials were discarded if they contained saccades in the first 200 ms after SPEM onset.

To detect if the recorded PCs demonstrated saccade-related SS responses, we compared the mean SS firing rate during the baseline period (100–300 ms before saccade onset) with the mean SS firing rate during 100 ms before until 200 ms after saccade onset for each of the 8 saccade directions using a Wilcoxon signed-rank test (*p* < 0.05). A PC SS unit was considered to be saccade-related if it showed a significant SS firing rate modulation in at least one saccade direction. In order to pinpoint the preferred direction of neurons, we fitted the plot of discharge rate as function of direction with a sine function. Neurons for which this fit was significant (χ^2^ test, *p* < 0.05) were considered in a subsequent analysis of direction preferences in the sample. PC SS units with saccade-related activity beyond or below baseline firing rate by 3 times standard deviation were classified as bursting or pausing units, respectively. If both significant bursting and pausing components were found in a PC unit, this unit was considered as biphasic and both components were considered independently in the later analysis of direction selectivity. For the bursting component the direction with the largest discharge as predicted by the sine fit was taken as the preferred one and correspondingly in the case of a pausing component the direction with the lowest predicted discharge. For units with only one component in the firing patterns, the angular distance between the preferred directions was calculated by comparing the preferred directions for saccades and SPEM based on that component. For units with two components during at least one type of eye movement, the angular distance was calculated by comparing the preferred directions based on the component(s) present in the responses to both types of eye movements.

To assess if PC SS units exhibited SPEM-related modulation we compared the SS firing rate during the baseline period with the SS firing rate in the period of 100 ms before and 200 ms after SPEM onset for each of the SPEM directions tested (Wilcoxon sign rank, *p* < 0.05)^24^. The preferred direction of those neurons, for which the fit was significant (χ^2^ test, *p* < 0.05), was computed separately for bursting and pausing components, which is similar to what we did for saccade-related neurons.

To analyse the relationship between the SS discharge rate (FR) and eye movement kinematics within a time period of −100 to +200 ms relative to movement onset, we fitted the average spike density function of individual PC SS units with a linear model with eye position (pos(t)), velocity (vel(t)) and acceleration (acc(t)) as independent variables.


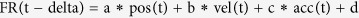


The coefficients a, b, c, d and the time shift delta coefficient in the above equation were chosen such as to maximize the coefficient of determination (CD), which indicates the goodness of the fit capturing the proportion of the variance in the dependent variable predicted from the independent variables. The time shift delta coefficient was restricted to values between −100 and +100 ms. To reveal the relative contributions of each kinematic variable, we omitted one or two variables in the equation and compared the resulting CD with the one for the complete fit. Cohen’s D^25^ was used to compare the quality of the fits between various models. It was obtained by dividing the difference between mean CDs of the two by the pooled standard deviation of their CDs. Cohen’s D values bigger than 0.8 indicate ‘large’ effects, whereas those smaller than 0.3 reflect ‘small’ effects. The remainder is considered as ‘medium’ effect.

## Results

### Sample of OMV PCs

**Figure 1:**
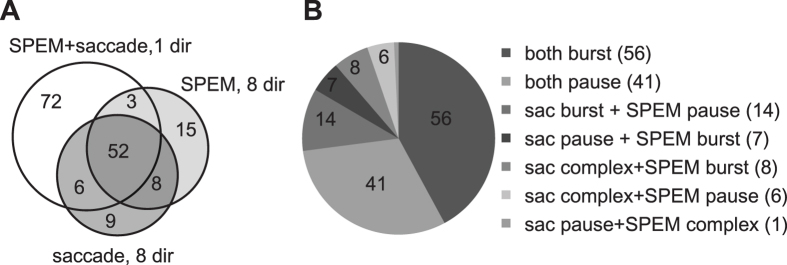
Breakdown of eye movement-related OMV PC SS units studied. (**A**) Venn diagram of units summarizing how many units subjected to various paradigms were considered. (**B**) The pie chart shows the numbers of units exhibiting specific firing pattern for saccades and SPEM. The number of units for each category is shown in brackets.

**Figure 2:**
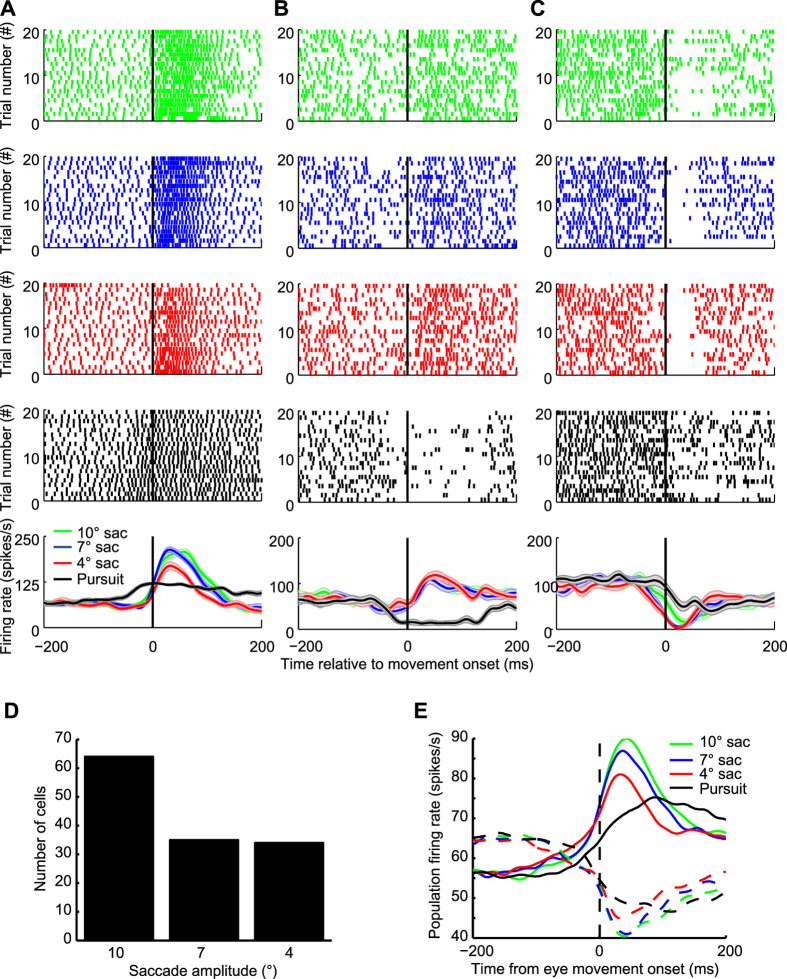
(**A–C**) Responses of three exemplary OMV PC SS units to saccades of different amplitudes and to SPEM in the same direction (A. 180° (left), B. 135° (upper-left), C. 0° (right)). The neuronal activity is characterized by raster plots (4 upper rows) and by mean spike density functions (last row) aligned to movement onset are plotted below the raster plot. 10°, 7°, 4° saccades and SPEM are represented in green, blue, red and black respectively. The partially transparent bands surrounding the mean spike density functions reflect the SEM. (**D**) Histogram of preferred saccades amplitudes based on n = 133 units. (**E**) Plots of population activity of all bursting units (n = 69, closed lines) and all pausing units (n = 64, dash line) for 10° (green), 7° (blue) and 4° (red) saccades. Population activity for SPEM is plotted in black.

We recorded a total of 165 PCs with eye movement-related SS discharge from the OMV (n = 92 in subject E and n = 73 in subject I) (Fig. 1A). In 72 out of the 165 units, the comparison was confined to one direction chosen by the experimenter as best direction based on subjective criteria (see Methods). The other 93 units could be tested for responses to saccades and/or SPEM for the full set of 8 directions in the frontoparallel plane. For a subgroup of 60 out of the 93 units complete direction tuning functions could be obtained for both types of eye movements. In the remainder it was restricted to either saccades (15 units) or SPEM (18 units) and supplemented by a single direction test of the respective other type of eye movement. 133 out of all 165 eye movement-related units (=81%) exhibited significant discharge modulation for saccades and SPEM in the same direction. As summarized in Fig. 1B the numbers of neurons with eye movement-related discharge increases, decreases and, occasionally, more complex profiles (i.e. increase–decrease or decrease–increase) was comparable in the 7 groups of units distinguished in the pie chart shown in Fig. 1A. As discussed in more detail further below, response types for saccades and SPEM were mostly, but not always, congruent (97 out of 133 units), meaning that PCs that for instance fired a burst in conjunction with saccades usually also exhibited a discharge increase during pursuit initiation (Fig. 2A).

Individual PCs SS could exhibit their highest (in the case of bursting units) or lowest (in the case of pausing units) firing rate for any of the three saccade amplitudes tested (Fig. 2A–C). However, more units preferred 10° saccades (see Fig. 2D). This population bias for larger amplitude saccades explains that also the population firing rates calculated separately for the subgroups of bursting and pausing units exhibited the strongest modulation for the largest saccade amplitude (Fig. 2E).

### PC SS encode eye movement kinematics

**Figure 3:**
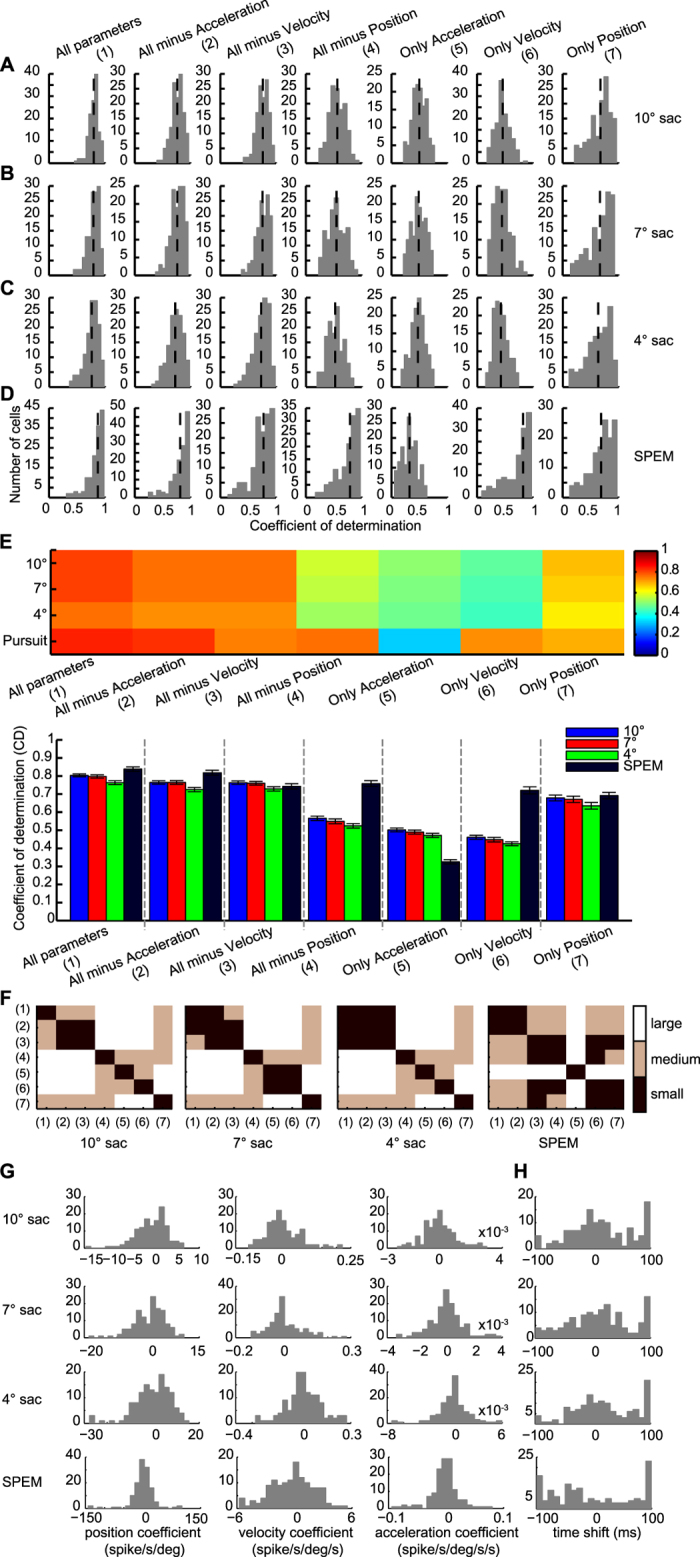
(**A–D**) Distribution of coefficients of determination (CDs) obtained by fitting discharge rates of individual OMV PC SS as function of the kinematic variables eye position, velocity and acceleration and different versions of slimmed down models for different amplitude saccades (**A–C**) and SPEM (**D**). The vertical dashed lines indicate the median CD for each model and paradigm. (**E**) The upper panel depicts a plot of mean CDs represented by different colors for the various models and paradigms also shown in A-D. The lower panel is a bar chart of the mean CDs. Error bars indicate SEM. Model types are identified by numbers shown in parentheses. (**F**) Plot of the size of the effect of moving from one particular type of model to another one indicated by the number as measured by Cohen´s D for 10°, 7°, 4° saccades (first three plots) and SPEM (plot on the most right). The three effect size categories (small, medium, large; see Methods) are color coded. (**G**) Distribution of position, velocity and acceleration coefficients. (**H**) Distribution of time shifts.

To study the relationship of eye movement kinematics and saccade- and SPEM-related responses respectively, we investigated how well the discharge of individual PCs could be predicted by a linear combination of eye acceleration, velocity and position independently for saccades and SPEM. To this end, the responses to saccades of 10°, 7° and 4° saccades and to SPEM were fitted separately, but for the same reference direction. This reference direction was the best direction for saccades as determined by a quantitative analysis of responses to saccades in 8 directions (58 units) or the preferred saccade direction as estimated subjectively by the experimenter, listening to the audiomonitor (75 units). Figure 3 summarizes the results obtained for the 133 units subjected to this analysis. As shown in the first three panels in column 1 of Fig. 3A–D, the mean coefficients of determination (CDs) were about 0.8 for both saccades, independent of amplitude, and for SPEM. In other words, the linear kinematic model was able to predict most of the variance in the PC simple-spike discharge rate based on the eye movement data. We next tried to estimate the contribution of each kinematic parameter by removing one out of the 3 kinematic parameters from the model or, alternatively, by keeping just 1 particular parameter. The distribution of CDs obtained after removal of eye acceleration, velocity or position respectively from the model is shown in columns 2–4 of Fig. 3A–D, whereas columns 5–7 present the CDs obtained when restricting the model to a single kinematic parameter. In the case of saccades, removal of any one or two kinematic parameters resulted in significant decreases of CDs (Friedman’s 2-way rank ANOVA comparisons of the CD distribution for the 3-parameter model with the CD distributions for any of the reduced models with the factors ‘model type’ and ‘saccade amplitude’, factor model type: *p* < 0.001 and factor saccade amplitude: *p* = 0.0074). However, the extent of these decreases was different as indicated by the plot of Cohen’s D for the various paired model comparisons shown in Fig. 3F: removal of eye acceleration or velocity reduced the CDs only slightly (Cohen’s D < 0.8) to a median of 0.76 in the case of acceleration and of 0.76 in the case of velocity. On the other hand, removing eye position from the model resulted in a substantially higher decrease of the CDs to a median CD of 0.54 (Cohen’s D > 0.8). These results indicate that eye position is the most important kinematic parameter encoded by PC SS during saccades. This conclusion is supported by constraining the linear model to one kinematic parameter only (see columns 5–7 in Fig. 3A–C). Restricting the model to eye position yielded a median CD of 0.71, only marginally smaller than the median CD obtained when applying the full-fledged model (Cohen’s D < 0.8). On the other hand, fits based on either acceleration or velocity yielded substantially smaller median CDs (Cohen´s D > 0.8, eye acceleration: median CD = 0.48, eye velocity: CD= 0.43). As shown in Fig. 3F, the pattern of effect sizes of the various model modifications was similar, which indicates that the pattern was independent of saccade amplitude.

Also fitting the PC SS responses to SPEM in the same direction as the one for saccades by the linear combination of all 3 kinematic parameters yielded a substantial explanation of the variance as indicated by a median CD of 0.89 (Fig. 3D). In the case of SPEM, removal of any parameter, except acceleration, resulted in a significant CD decrease (*U*-test, *p* < 0.05). The analysis of Cohen’s D (Fig. 3F, panel 4) demonstrated that removal of eye velocity and eye position had a medium effect (0.3 < Cohen’s D < 0.8), causing a decrease of CD to 0.79 and 0.83, respectively. On the other hand, removal of eye acceleration had weaker effects as indicated by small Cohen’s D (Cohen’s D < 0.3) (acceleration removal: median CD = 0.87, *U* test, *p* = 0.29). Correspondingly, restricting the model to velocity caused only a weak drop of CDs to a median CD of 0.81; 0.3 < Cohen’s D < 0.8). Restricting the model to eye position caused a modest decrease to a median CD of 0.74 (0.3 < Cohen’s D <0.8) and finally, restricting the model to eye acceleration resulted in a substantial decrease to a median CD = 0.32; Cohen’s D > 0.8). Figure 3E provides a visual summary of the modeling results discussed before. Figure 3G depicts the distributions of the position, velocity and acceleration coefficients for the two types of eye movements. Figure 3H shows the distribution of the time shifts (delta) between the eye movements and the discharge giving the best fits for both saccades and SPEM. The fact that the delta for saccades peaked around 10 ms indicates that the discharge usually lagged the saccade. In contrast to the distribution for saccades the one for SPEM was rather flat, reflecting a mixture of leading and lagging responses. Note that occasionally the best fits were found for latencies at the boundaries of the range of ±100 ms, both for saccades as well as for SPEM. However, the pattern of CDs obtained did not change significantly when excluding these odd cases (see Supplementary Fig. S2). Consistent with a previous study of abducens neurons, a negative correlation was found between position and velocity coefficients and eye velocity^26^. The aforementioned analysis was based on regressing discharge as a function of movement kinematics within a window of −100 to 200 ms relative to eye movement onset. One may wonder if the results depended on the choice of the window. All in all, this is not the case. As summarized in Supplementary Figs S3 and S4, the basic pattern of the relative weights of acceleration, velocity and position was the same when choosing a narrower window. However, the choice of the window affected the delta, i.e. the latency between discharge and the eye movement as the clear peak in the distribution indicating that on average discharge lagging saccades was no longer visible when choosing shift ranging from −50 to 50 ms and data from 50 ms before and 100 ms after movement onset (see Supplementary Fig. S3).

Taken together, the results clearly show that the PC SS firing patterns can be well explained by the linear model. Although all three kinematic parameters make contributions to an explanation of the discharge variance, their relative weights are not the same for the two types of eye movements compared. Rather the weight profiles depend on the type of goal directed eye movement considered. Whereas saccade-related responses are dominated by eye position, those to SPEM are mostly dependent on eye velocity and eye position.

### Comparison of directional preferences of eye movement-related PC SS units for saccades and SPEM

**Figure 4:**
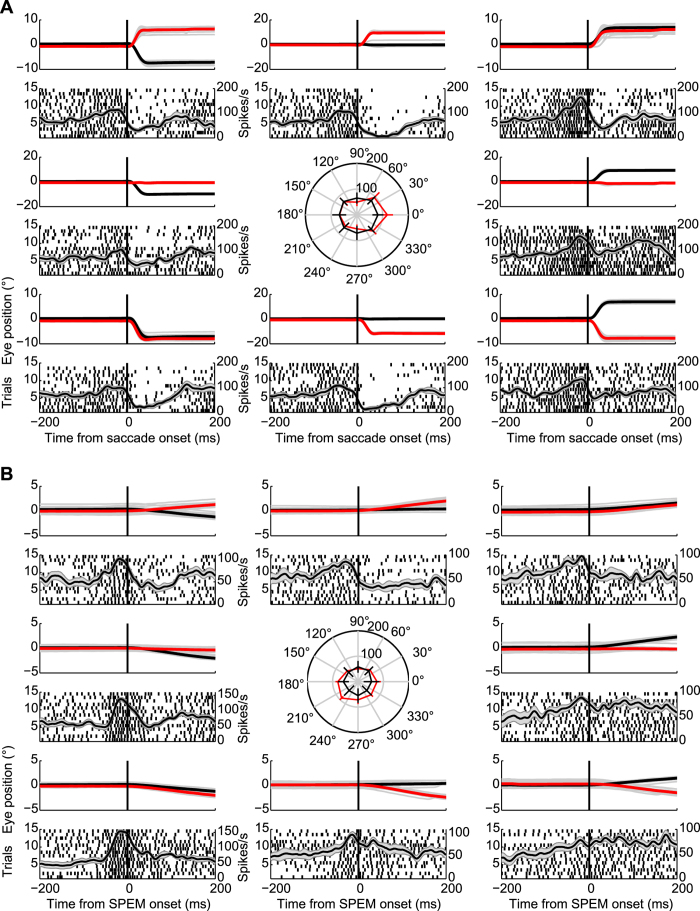
Responses of exemplary OMV PC SS to saccades (**A**) and SPEM (**B**) made in 8 directions in the frontoparallel plane. The top traces in each panel depict the mean horizontal (black) and vertical (red) eye positions with gray lines indicating the eye position for each trial. Neural activity is represented underneath the eye movements records by raster plots aligned to saccade onset and spike density functions plotted on top of the raster plots. The grey bands underneath the spike density functions characterize the SEM. The central panels in A and B present polar plots of mean firing rates plus standard deviation as function of direction.

**Figure 5:**
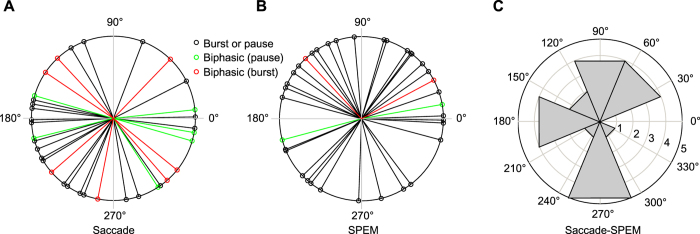
Polar distribution of the eye movement directions exhibiting the most vigorous firing for each neuron. (**A**) Polar distribution of preferred saccade directions (see Methods, Results for details) based on n = 41 units. Units with only one firing component are shown in black and preferred directions of the pausing and bursting components of units with two firing components are displayed in green and red, respectively. (**B**) Polar distribution of preferred SPEM directions of n = 38 units tested for SPEM-related responses. (**C**) Polar distribution of angular distance between preferred saccade and SPEM directions for the n = 21 units for which complete direction tuning data were available for both saccades and SPEM.

Figure 4A and B depict the responses of an exemplary PC SS unit to saccades and SPEM in 8 directions, respectively. This neuron exhibited higher firing rate for rightward saccades and lower left SPEM. To compare the directional preferences of PC SS units for saccades and SPEM, we plotted the mean saccade- and SPEM-related discharge rates as function of the 8 directions and fitted sine wave functions to the plots (see Methods for details). Figure 5 depicts the distribution of preferred directions for saccades (Fig. 5A) and SPEM (Fig. 5B). For the subset of 21 units for which preferred directions could be determined for both saccades and SPEM, we calculated the angular differences between the preferred directions for the two types of eye movements for each individual unit. Figure 5C shows the distribution of these differences. It clearly indicates a lack of relationship between preferred directions for the two types of eye movements: no unit exhibited identical preferred directions for saccades and SPEM and overall angular differences were distributed uniformly over all 4 quadrants (Rayleigh test, *p* = 0.61).

The kinematic analysis discussed above was confined to saccades and SPEM made in one and the same direction. We wondered if the kinematic preferences of PC SS units suggested by the 1-direction approach would remain valid for other directions as well. To obtain an answer we subjected the units for which datasets for all 8 directions were available for saccades (75 units) or for SPEM (78 units; intersection between the two 60 units) to a multi-linear regression analysis of discharge as function of eye movement kinematics. The analysis was carried out separately for saccades and SPEM and for each direction.

**Figure 6:**
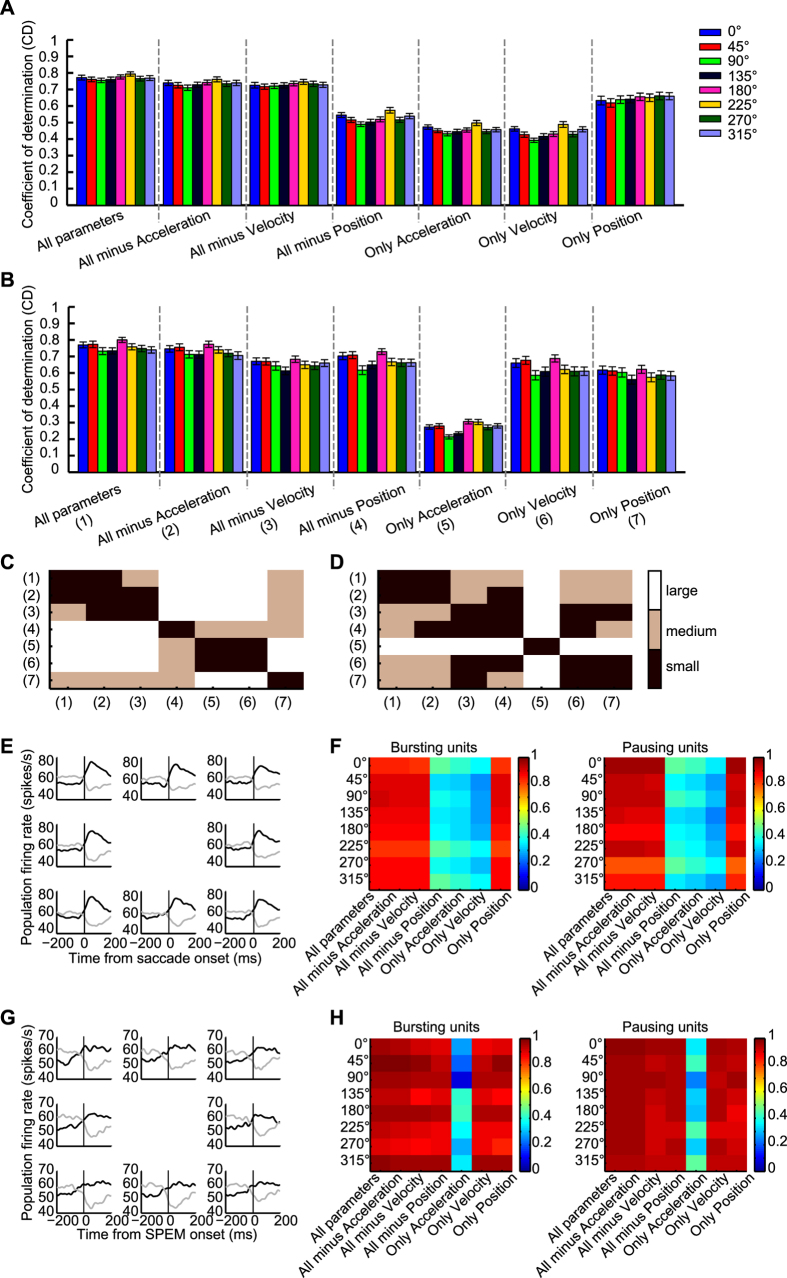
Distribution of coefficients of determination (CDs) obtained by fitting the population discharge as a function of the kinematic variables eye position, velocity and acceleration and different versions of slimmed down models for the 8 directions in the frontoparallel plane. (**A**) presents the CDs for the various variable constellations and the 8 directions for all PC SS tested for saccades. (**B**) all PC units tested for SPEM. Error bars in (**A**) and (**B**) indicate SEM. (**C and D**) Plots (**C**), saccades; (**D**) SPEM) of the size of the effect (measured by Cohen’s (**D**) of moving from one particular type of model to another one. The model types compared are indicated by the numbers on the two axes. The plots are based on eye movements to 0° serving as reference direction. The three effect size categories (small, medium, large; see Methods) are color coded. (**E**) The left panel depicts the population spike density functions aligned with saccade onset separately for bursting units (n = 40) and for pausing units (n = 35) for the 8 directions tested. (**F**) Plots of CDs represented by different colors for the various models and directions. The panel on the left represents all bursting units; the one on the right represents all pausing units. (**G and H**) Population activity for SPEM in 8 directions and corresponding CDs (35 bursting units, 43 pausing units). Format of presentation is the same as for saccades in (**C** and **D**).

Figure 6 plots the mean CDs as function of direction for the 3 parameter model and the various 2 and 1 parameter models as described earlier for saccades (A) and for SPEM (B). The results are in line with the ones obtained for the units that were tested for one direction only. Based on a linear combination of the three kinematic parameters, the PC SS discharge could be reliably predicted as indicated by median CDs > 0.75 with eye position being the dominant parameter in case of saccades and eye velocity plus a weaker contribution of eye position in the case of SPEM. Removal of any one or two kinematic parameters resulted in a significant decrease of CDs (*U* test, *p* < 0.001), except the removal of acceleration in case of SPEM (*U* test, *p* = 0.13). Importantly, the CD patterns for different directions were quite similar (correlation r > 0.9, *p* < 0.001), indicating that the kinematic profiles of individual units were independent of eye movement direction. The effect sizes as gauged by Cohen’s D, calculated for a ‘reference’ direction of 0° for saccades and SPEM are summarized in Fig. 6C and D, respectively. The pattern shown is very similar to the one yielded by the analysis of the sample of units tested for various saccade amplitudes but for only one direction of eye movements summarized in Fig. 3F.

Finally, we calculated the collective instantaneous discharge rate, separately for each direction and separately for saccades and SPEM for the same units considered before. Figure 6E,G plot the resulting collective discharge rate as function of time relative to the onset of the movement (E: smooth pursuit, G: saccades) separately for units with burst and pause responses. The panels on the right plot the CDs as function of direction for bursting units and pausing units respectively. In Fig. 6F,H we summarized the results obtained when subjecting the population discharge to the same multiple linear regression as used for the modelling of individual units. The modelling results for the population discharge were fully consistent with the results for individual units, the only difference being that the CDs (median CD > 0.87) were significantly higher than those obtained based on individual units (median CD about 0.75; *t*-test, *p* < 0.001). This is not surprising given the fact that averaging neuronal responses and eye movement data will lower the variance.

## Discussion

The aim of this study was to compare the sensitivity of PC SS to saccades and to SPEM. The reason for this interest was the increasing evidence - discussed in the introduction - for a role of the OMV in both types of goal directed eye movements, including anecdotal descriptions of some PCs SS responding to both saccades and SPEM^18^,^19^. Actually, we now found that the overwhelming majority of PC SS units in the OMV are ‘dual’ PCs responding to both types of goal-directed eye movements. The discharge associated with saccades as well as with SPEM depended on the kinematics of the eye movements made. However, the kinematic variables of particular significance for the prediction of spike trains differed for the two. Whereas both saccade- and SPEM-related responses were influenced by all three kinematic variables considered in a multiple linear regression of discharge rate as function of eye movement kinematics, the relative weights of the kinematic variables depended on the type of goal directed eye movement performed. In the case of saccades, eye position was the most important kinematic variable, whereas it was eye velocity in the case of SPEM. These distinct kinematic profiles were independent of eye movement direction in the frontoparallel plane. Individual PC SS units not only exhibited different kinematic preferences for saccades and SPEM but, surprisingly, also unrelated directional preferences for eye movements in the frontoparallel plane.

The dependence of SPEM responses of dual OMV PC SSs on eye velocity is in line with previous reports on the kinematic preferences of OMV PCs, tested for SPEM only^22^,^24^. However, one might think that the strong influence of eye position in the case of saccades is at odds with a previous report^27^ emphasizing a reflection of eye speed and direction in the population discharge of OMV PCs. However, closer consideration indicates that there is actually no contradiction whatsoever. On the one hand, our kinematic analysis also showed that eye velocity mattered, albeit to a less extent than eye position. On the other hand, we have previously demonstrated that the OMV PC SS population discharge predicts saccade duration, which in turn is tightly correlated with saccade amplitude^28^,^29^. In other words, a strong influence of eye position had to be expected. The notion that the OMV controls eye position in the case of saccades and eye velocity in the case of SPEM is in good accordance with key assumptions of well-established models of saccade and SPEM generation respectively, reflecting the need to integrate latency-free estimates of relevant state variables. In the case of saccades, the state variable emphasized is current eye position that is compared with desired target location, the difference of the two telling the system how much further to move the eyes^30^,^31^. In the case of SPEM, models usually build on a prediction of eye velocity, avoiding the detrimental delay of visual feedback on the pursuit eye movement^32^,^33^.

Optimal saccade and SPEM performance requires short-term calibration of these state variables, an adjustment that is known to depend on the integrity of the cerebellum^10^,^11^,^14^,^34^ and the adjustment of the OMV SS output^24^,^35^. The evidence available suggests that the learning-based adjustments of saccade amplitude are a consequence of error-based changes of the synaptic weights of parallel fiber (PF) synapses^36^,^37^. Most probably, the same holds for learning-based changes of SPEM velocity. We have recently obtained preliminary behavioral evidence suggesting that learning-based adjustments of one type of goal-directed eye movement do not spill over to the other one. Such a high degree of specificity is surprising if – as shown by the study at hand – OMV PCs are a common node shared by the two pathways for saccades and SPEM. How can specificity of learning be maintained although information on the two types of eye movements converges on individual OMV PCs? If we assume that both saccadic and SPEM learning are the result of changes of the strength of parallel fiber synapses due to an interaction between PF signals and error information conveyed by the climbing fiber system, one likely answer is that the OMV PCs must reserve individual synapses for the one or the other type of goal-directed eye movement. The duality of OMV PCs might pose a second problem, though, namely the ambiguity of their output signals which is not resolved at the level of the caudal fastigial nucleus (cFN). The latter conclusion is based on our recent observation that also cFN eye movement-related neurons are predominantly dual, i.e. driven by both saccades and SPEM^38^. In other words, the premotor and motor brainstem machinery for saccades and smooth pursuit will have to deal with cerebellar information not unambiguously associated with the one or the other type of eye movement. Hence, if the brainstem machinery were organized in an eye movement type-specific way, PC activity related to eye movement type A should exert a spurious influence on the brainstem center for eye movement type B. In order to avoid the activation of the inexpedient eye movement B, the type A-related PC signal would have to be thwarted at the center for B by the absence of direct, extracerebellar input related to eye movement B. This complication would only be avoided if SPEM and saccades shared a common brainstem pathway. If this is the case is unclear. It is usually assumed that the same motoneurons support saccades and SPEM and in general different types of eye movements^6^,^7^. However, anecdotal physiological observations^39^ and anatomical studies^40^,^41^ have suggested that the eye muscle fibers and the motoneurons that support them show heterogeneity related to different types of oculomotor behaviors. On the other hand, the immediate premotor targets of cFN axons, like the paramedian pontine reticular formation (PPRF), the rostral interstitial nucleus of the medial longitudinal fasciculus (riMLF), the central mesencephalic reticular formation (cMRF), the perihypoglossal nucleus (PHN) and the pontine raphe (PR)^42^, are commonly discussed as saccade-specific. However, at least in the case of the omnipause neurons, located in the PR, there is evidence that the discharge is not only suppressed by saccades but also inhibited to some extent during SPEM^43^. Also neurons in the PPRF, the riMLF and the cMRF have been shown to contain neurons that are both saccade- and pursuit-related^44^,^45^,^46^,^47^. Hence, although much of the evidence available seems to be in line with the notion that the pathway downstream of the OMV shares the duality of the OMV output, this question cannot be conclusively answered. Therefore it remains open if the organization of the downstream pathway is such as to make additional circuitry for the identification of the type of eye movement at stake dispensable. Irrespective of the question if these thoughts on the wiring of input and output of dual OMV PC are close to reality or not, the convergence of saccade- and SPEM-related signals at the level of single OMV PCs probably comes with costs. Hence, what could the gain be that outweighs these costs? An obvious answer is that convergence allows the cerebellum to accommodate two types of eye movements with one set rather than two sets of PC. Yet, this answer can only satisfy if the additional investments needed in order to disentangle the saccade and SPEM signals downstream of the OMV PCs will indeed be dispensable.

## Additional Information

**How to cite this article**: Sun, Z. *et al*. The same oculomotor vermal Purkinje cells encode the different kinematics of saccades and of smooth pursuit eye movements. *Sci. Rep.* **7**, 40613; doi: 10.1038/srep40613 (2017).

**Publisher's note:** Springer Nature remains neutral with regard to jurisdictional claims in published maps and institutional affiliations.

## Supplementary Material

Supplementary Information
