# Supplementary material for: The same oculomotor vermal Purkinje cells encode the different kinematics of saccades and of smooth pursuit eye movements
Source: Sci Rep. 2017 Jan 16;7:40613. doi: 10.1038/srep40613 (PMC5238383; doi:10.1038/srep40613)
Supplement: Supplementary Information [file srep40613-s1.pdf]

The same oculomotor vermal Purkinje cells encode the different kinematics of saccades and of smooth pursuit eye movements

Zongpeng Sun[1,2], Aleksandra Smilgin[1,2], Marc Junker[1,2], Peter W. Dicke[1], Peter Thier[1]*

[1] Department of Cognitive Neurology, Hertie Institute for Clinical Brain Research, University of Tübingen, 72076 Tübingen, Germany
[2] Graduate School of Neural and Behavioural Sciences, International Max Planck Research School for Cognitive and Systems Neuroscience, University of Tübingen, 72074 Tübingen, Germany

* To whom correspondence should be addressed:
Peter Thier, Department of Cognitive Neurology, Hertie Institute for Clinical Brain Research, Hoppe-Seyler-Str. 3, 72076 Tübingen, Germany, E-mail: thier@uni-tuebingen.de.

Number of supplementary figures: 4

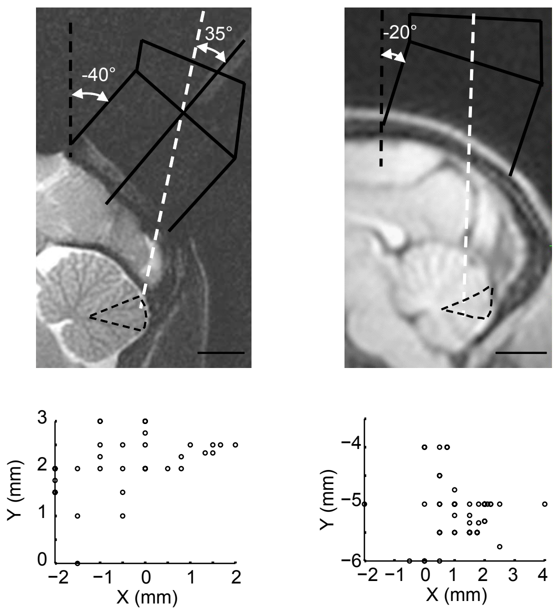

Supplementary Fig. S1: Sagittal MRI view showing the recording chamber and its orientation relative to the brainstem in monkeys I and E (left panel and right panel, respectively). The locations of recorded PC units in the OMV are shown in the scatter plots at the bottom. The dashed lines delineate the oculomotor vermis. The white dashed lines indicate the track of an electrode entering the center of the chamber. Scale bar, 10 mm.

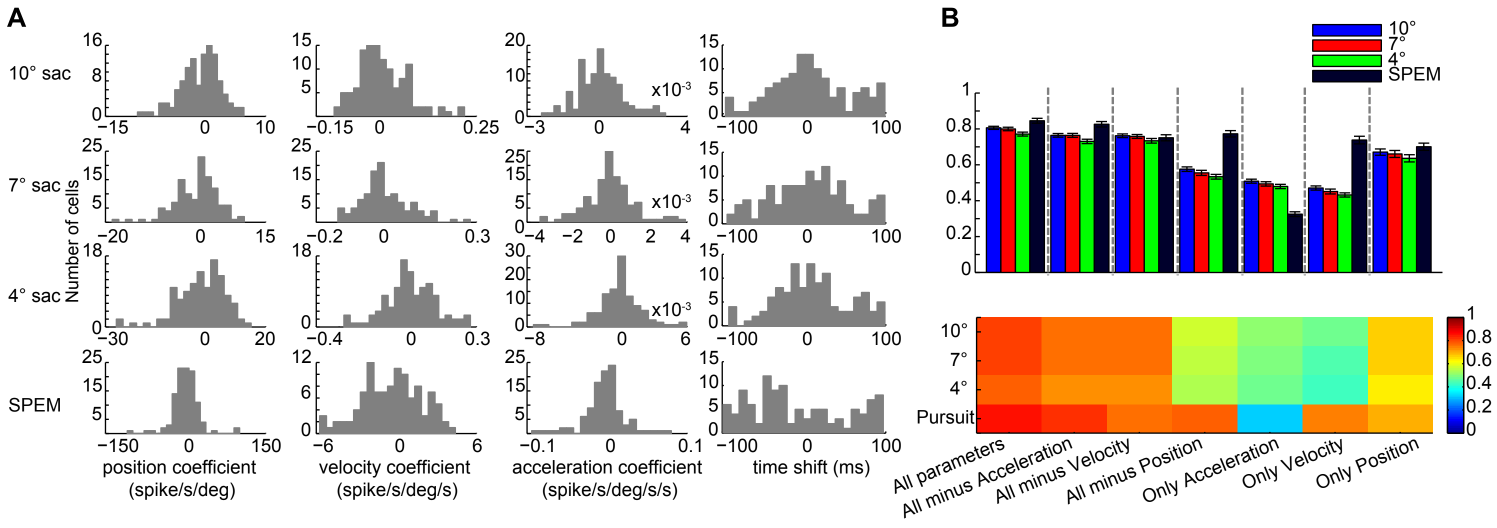

Supplementary Fig. S2: A. Distribution of position, velocity and acceleration coefficients and distribution of time shifts based on the analysis using a shift ranging from -100 ms to 100 ms, after removal of fits that involved optimal time shifts corresponding to the extremes of the shift window. The first three rows display the plots for 10°, 7° and 4° saccades. The last row presents the plot for SPEM. The distribution of position, velocity and acceleration coefficients is shown in the first 3 columns and the distribution of time shifts in the last column. B. The upper panel is a bar chart of the mean CDs. Error bars indicate SEM. The lower panel depicts a plot of mean CDs represented by different colors for the various models.

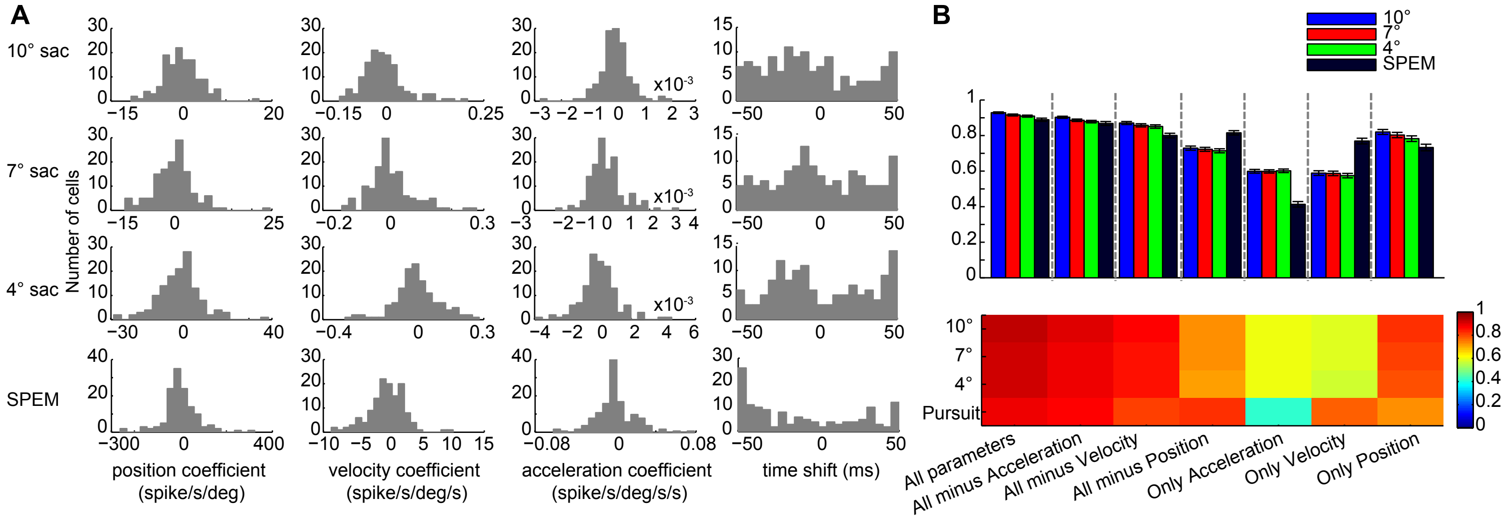

Supplementary Fig. S3: A. Distribution of position, velocity and acceleration coefficients and distribution of time shifts based on the analysis using a shift ranging from -50 ms to 50 ms and data from 50 ms before and 100 ms after movement onset. The first three rows display the plots for 10°, 7° and 4° saccades. The last row presents the plot for SPEM. The distribution of position, velocity and acceleration coefficients is shown in the first 3 columns and the distribution of time shifts in the last column. B. The upper panel is a bar chart of the mean CDs. Error bars indicate SEM. The lower panel depicts a plot of mean CDs represented by different colors for the various models.

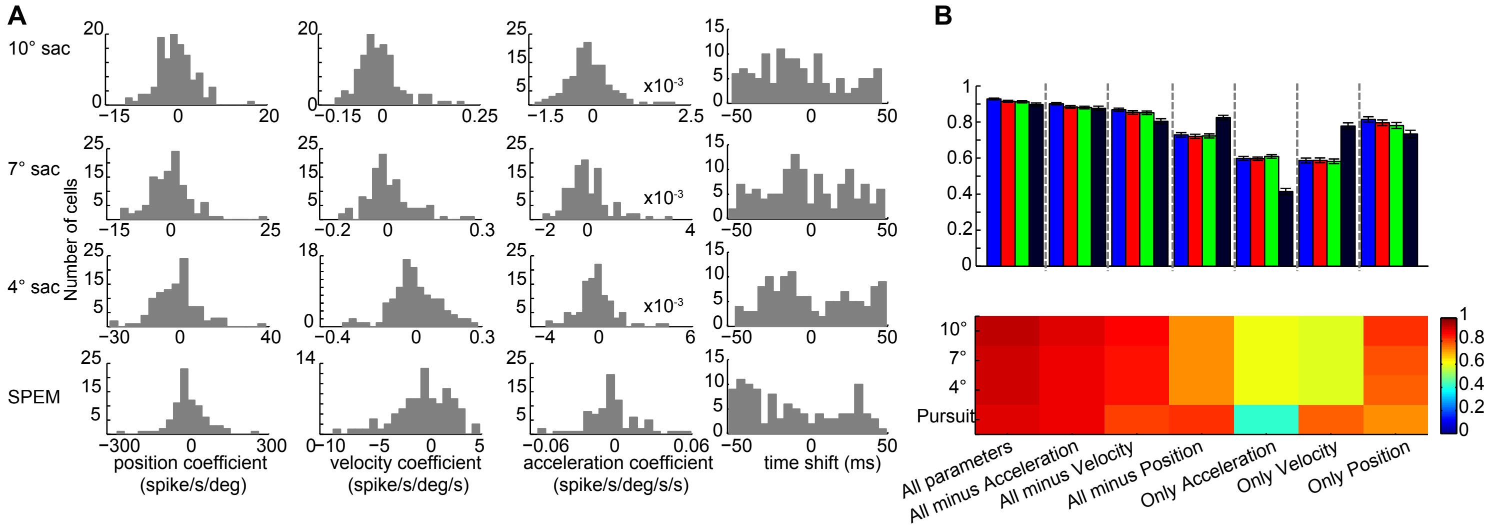

Supplementary Fig. S4: A. Distribution of position, velocity and acceleration coefficients and distribution of time shifts based on analysis using shift ranging from -50 ms to 50 ms and data from 50 ms before and 100 ms after movement onset, after removal of fits that involved optimal time shifts corresponding to the extremes of the shift window. The first three rows display the plots for 10°, 7° and 4° saccades. The last row presents the plot for SPEM. The distribution of position, velocity and acceleration coefficients is shown in the first 3 columns and the distribution of time shifts in the last column. B. The upper panel is a bar chart of the mean CDs. Error bars indicate SEM. The lower panel depicts a plot of mean CDs represented by different colors for the various models.
